# Patient-Reported Outcomes Measures in Abdominal Cancer Surgery and Student-Led Surgical Research

**DOI:** 10.1245/s10434-021-09686-5

**Published:** 2021-03-31

**Authors:** Augusto León, Klaus Puschel, Augusto E. León, Francisca Honold

**Affiliations:** 1 Program of Cancer, Department of Surgical Oncology, Catholic University of Chile, Santiago, Chile; 2 Program of Family Medicine, Catholic University of Chile, Santiago, Chile; 3 Resident of Radiotherapy, Instituto de Radiomedicina IRAM, Diego Portales University, Santiago, Chile; 4 Resident of Surgery, University of Chile, Santiago, Chile

In this issue of the *Annals of Surgical Oncology,* Mihaljevic and coauthors show the importance of patient-reported outcomes measures (PROM) in abdominal cancer surgery,^1^ while also offering an excellent example of student-driven, surgical research and stressing the importance of integrating medical students in a clinical trial.

## The Importance of PROM

Patient-reported outcome measures have emerged during the past decade as a powerful tool for directly capturing patients’ perspectives and stimulating their autonomy in the decision-care processes.^2^ PROM can differ significantly from outcomes reported by clinicians and are frequently worse than the latter. This also is true regarding adverse effects (AEs), for instance severity of postoperative pain, which are usually underscored by clinicians compared with patients. Even in the more controlled environment of a clinical trial setting, up to 50% of AEs can go unreported.^3^

PROMs are an objective measurement tool, even if the outcome they quantify is somewhat subjective; they undergo a rigorous development and validation process in order to make them an objective tool, with reproducible results from which valid conclusions can be drawn.^4^ Major cancer organizations, including the Food and Drug Administration and the National Cancer Institute, have made strong recommendations for the inclusion of a PROM approach in routine clinical cancer care.

PROMs can help to attain a more adequate care of cancer survivors, whose needs often can go unrecognized and unsatisfied.^5^ As shown by Dueck et al. (2015), the Patient-Reported Outcomes version of the Common Terminology Criteria for Adverse Events (PRO-CTCAE) improves the precision and reliability of the information gathering regarding AEs. The data metrics of reported AE allow for improvements to patients’ quality of care, rational funding of clinical research, better design of public health policy, etc.^3^

The relevance of the PROM approach in cancer arises from the strong evidence showing the significant physical and psychosocial consequences that cancer treatment can have on patients’ QOL. These sequelae can often go unrecognized by health care teams.^6^,^7^ Our group found that 30% of breast and colon cancer survivors treated at a leading Cancer Center in Latin America had significant psychosocial distress, yet only a third of them were assessed for said factors by their healthcare teams.^8^ These results are very similar to those reported by other oncology groups.^6^ PROM offers an opportunity to address those key psychosocial factors in a systematic patient-based approach.

Similar to most studies published on PROM and cancer care, Mihaljevic et al. found a lack of correlation between postoperative complications or death and any of the PROM items evaluated.^2^ These results could reflect the different perspectives of health and disease assessed when evaluating QOL, postoperative complications, or death. For example, a patient might have a minor postoperative complication but a major decrease in QOL associated with financial difficulties due to cancer treatment. On the other hand, a patient might strongly improve their QOL after cancer surgery but die from a myocardial infarction in a subsequent stage of their cancer treatment.

The newly developed PRO-CTCAE and Computerised Adaptive Testing (CAT) EORTC quality of life questionnaire (QLQ-C30) tools tested in the study followed the same trend as the HRQoL tool used for evaluating quality of life after major abdominal cancer surgery in this group of patients. These results are encouraging, showing that new practical tools for estimating quality of life dimensions reported directly from the patients are available for surgical cancer patients.

The digital patient-reported outcomes has some limitations that can be divided in four categories: ability to use PROMs, engagement, emotional distress, and technical barriers.^9^ Some reasons for nonparticipation can be related to fear or expectations; these can be addressed by providing the patients with adequate information, identifying possible causes for concern. This makes adequate communication between patient and clinician of great importance. Ability to use PROs has to do with the fact that patients with long-term diseases can lack the energy and interest to undertake answering the questionnaires. The periodicity with which tests are applied, and their length, should be correlated with the dropout rate. Repetitive or overly detailed questions concerning symptoms and general health may cause anxiety and dissatisfaction in some patients. There also may be concerns about data privacy and frustration with the hardware or software implementation.

## The Importance of Integrating Medical Students in a Clinical Trial

The PATRONUS study conducted by Mihaljevic et al. is very welcome, because it delivers a strong message on the relevance of empowering the main players of academic medicine and clinical practice, i.e., students and patients. The CHIR-Net Sigma study group is composed of medical students interested in the generation of new knowledge to improve clinical care through studying the direct experience of patients, making patients the protagonists of their own care process.

Empowerment of students and patients is the key common ingredient of the student-centered learning and patient-centered medicine approaches. Both of these have shown improved outcomes in medical education and medical care compared with traditional models.^10^,^11^

The development of research competences have been considered a key dimension of excellence in medical education.^12^ The main components of scientific research: curiosity, critical appraisal, methodology, team work, management, and honesty also are essential for the development of professional identity in medical students.^13^ The experience of PATRONUS, a multicenter, student-led, clinical study in Germany, is a great example of how to implement a concrete strategy to boost those values among medical students. The responsibility of coordinating more than 100 students from 15 different sites to work on 1 study protocol is a great example of teamwork, management, and persistence, surely combined with a large dose of enthusiasm and friendship among students. The academic surgeons who guided the experience are also an example of how to apply a student-centered learning approach. We believe the PATRONUS initiative to be a useful model for creating new, much-needed scientific collaborative networks.
